# Content of toxic and essential metals in recrystallized and washed table salt in Shiraz, Iran

**DOI:** 10.1186/2052-336X-12-10

**Published:** 2014-01-07

**Authors:** Mohammad Hassan Eftekhari, Seyed Mohammad Mazloomi, Marzieh Akbarzadeh, Mojdeh Ranjbar

**Affiliations:** 1 Department of Clinical Nutrition, School of Nutrition and Food Sciences, Research Center for Health Sciences, Shiraz University of Medical Sciences, Shiraz, Iran; 2 Department of Food Hygiene and Quality Control, School of Nutrition and Food Sciences, Shiraz University of Medical Sciences, Shiraz, Iran; 3 Department of Environmental Health Engineering, School of Health, Shiraz University of Medical Sciences, Shiraz, Iran

**Keywords:** Heavy metal, Sodium chloride, Wash

## Abstract

**Background:**

Table salt is the most commonly used food additive. Since most of the salt consumed in Iran comes from mines, contamination with heavy metals is a health concern. The commonest salt purification method in Iran is washing with water. But recently, some industries have turned to recrystallization method. The present study aimed to determine the level of essential and non-essential heavy metals in the table salt refined with recrystallization and washing methods.

**Methods:**

Thirty eight pre-packed salt samples were directly collected from retail market in Shiraz (22 samples refined with recrystallization method and 16 with washing method). The level of lead, cadmium, copper, zinc, nickel and cobalt was determined using Voltammetric method. Daily intakes of lead and cadmium as well as their weekly intakes were calculated.

**Results:**

The levels of lead, cadmium, copper, zinc, nickel and cobalt in recrystallized samples were 0. 30 ± 0.26, 0.02 ± 0.02, 0.11 ± 0.06, 0.34 ± 0.22, 0.15 ± 0.19 and 0.008 ± 0.007 μg/g, respectively, and also 0.37 ± 0.27, 0.017 ± 0.021, 0.19 ± 0.18, 0.37 ± 0.20, 0.13 ± 0.23 and 0.037 ± 0.06 μg/g in washed salt samples. The calculated weekly intake of lead and cadmium was 0.216 and 0.014 μg/kg, respectively for the recrystallized and 0.2653 and 0.0119 μg/kg for the washed salts.

**Conclusion:**

All values for toxic metals were lower than the permitted maximum for human consumption as prescribed by Codex and Institute of Standards and Industrial Research of Iran. Only 0.8652-1.0612% of lead and 0.17-0.2% of cadmium PTWIs are received via salt consumption weekly.

## Introduction

The polluted environment surrounding us exposes humans to different toxic substances leading to several complex disorders such as cancer, cardiovascular, respiratory and some chronic diseases [1]. The most common pollutants and also the most toxic ones are air pollutants and contaminants found in drinking water and foodstuff [1].

Human exposure to environmental chemicals occurs spontaneously through different sources [2]. For non-occupationally exposed humans, water and food consumption is the major route of exposure to toxic elements accounting for more than 90% of exposure to these substances, compared to that from inhalation and dermal contacts [3,4]. Chemical products and heavy metals are hazardous substances found in foodstuff [5].

Nowadays, food products’ contamination with heavy metals is an inevitable problem. Air, soil and water pollution leads to presence of these harmful elements such as lead, cadmium, mercury and arsenic in foodstuff [2]. Heavy metals are toxic to living organisms because they tend to accumulate in special tissues and contribute to several disorders [2].

Some elements such as lead, cadmium, mercury and arsenic are non-essential and are toxic even at low concentrations. Others such as copper and zinc are essential for humans; they play important roles in biological systems. When their intake exceeds special levels, they make harmful and toxic effects [6-9]. These non-essential heavy metals are toxic to cardiovascular, neural, hematopoietic, immunological and gastrointestinal systems [10-13]. They can also cause kidney dysfunction, anemia, liver toxicity, cancer and Alzheimer disease [10,12-15]. Because of chemical similarities, these heavy metals compete with essential elements and interact with several divalent transporters leading to disruption in different physiologic functions [10].

Table salt is one of the most commonly used food additives. In addition to salty flavor, it is used as flavor enhancer and also as a food preservative to inhibit the growth of spoilage and pathologic bacteria [9]. Since most of the salt consumed in the globe comes from mines, it is expected that heavy metal contamination is a serious concern about table salt [9]. Table salt is consumed daily, so any contamination, even at low levels, could be hazardous to the consumer’s health. Therefore, the concentration of heavy metals should be carefully monitored in this food additive.

Most of the salt consumed in Iran comes from mines, but a small proportion comes from solar evaporation of saline sea water [16]. Usually two purification procedures are used to remove impurities from rock salt. One of these procedures is washing with water [17]; the other is recrystallization [18], and recently Salex method has been used [19]. The most commonly used system in salt refining in Iran is washing with water [20,21]. But nowadays, the number of salt brands refined with recrystallization method is increasing in the market. In recrystallization process, a brine solution is treated with chemicals that precipitate most impurities (largely magnesium and calcium salts). Multiple stage evaporation is then used to collect pure sodium chloride crystals [22].

Since there are limited data on the level of heavy metals in recrystallized and washed table salts, the present study aimed to determine the level of essential and non-essential heavy metals in the table salt refined with recrystallization and washing methods.

## Materials and methods

Thirty eight pre-packed salt samples were directly collected from retail market in Shiraz (22 samples refined with recrystallization method and 16 with washing method). The level of lead, cadmium, copper, zinc, nickel and cobalt was determined using voltammetric method.

For the voltammetric measurement, a Polarograph/Voltammetr instrumental Model 797 VA Computrace (Metrohm, Swiss) was used interfaced with a hanging mercury dropping electrode (HDME). Stripping was performed with a Teflon coated bar at about 400 rpm using a magnetic stirrer (KIKA Labortechinik, Germany), Ag/AgCl (in saturated KCl) reference electrode, and auxiliary electrode of a platinum wire. At the beginning of the experiment, the solutions were deoxygenated with high purity nitrogen for 5 min, whereas before each analysis the solution was deoxygenated for 2 min.

### Voltammetric measurement

Five grams of the sample salt was first dissolved in 50 ml water. Then, 10 ml of the sample was transferred into the voltammetric cell containing 1ml of acetate buffer (pH = 4.6). The solution in the cell was aerated for 5 minutes by purging pure nitrogen gas. Anodic stripping was performed in differential pulse mode after selecting pre-concentration time of 180s, a scan rate of 4 mV/s and pulse amplitude of 50 mV. The concentrations of all the metals in the samples were determined using standards addition method on the Hanging Mercury Dropping Electrode (HDME) by means of Anodic Stripping Voltammetry (ASV).

The dietary intake of the studied heavy metals was also estimated and their associated risks were studied by comparing to the provisional tolerable weekly intakes (PTWIs). Considering the average daily salt intake to be 6 g/day [23-25], PTWI was calculated as follows:

$$\begin{array}{l} \text{Daily intake of heavy metals}\\ \;=\text{concentration of heavy metals in the salts}\\ \;\times \text{mean salt intake}\;\left(g/\text{person}/\text{day}\right) \end{array}$$

[23]

$$\begin{array}{l} \text{Weekly intake of heavy metals}\\ \;=\text{daily intake}\times \text{seven days}/\text{week} \end{array}$$

[23]

$$\begin{array}{l} \text{Weekly intake per body weight}\;\left(\text{kg}\right)\;\left(\text{PTWIs}\right)\\ \;=\text{weekly intake}÷\text{reference body weight}\;\left(60\;\text{kg}\right) \end{array}$$

[23,26]

The percentage of PTWI via salt consumption was also calculated.

### Statistical analysis

Data processing and analysis were done using SPSS, version 17 for windows (SPSS Inc, Chicago, USA). All data were expressed as mean (±Standard Deviation). We compared the metal contents of recrystallized and washed salts using Mann–Whitney U test. Significance level was set at p < 0.05.

## Results and discussion

Samples of salt refined with recrystallization and washing method were analyzed for lead, cadmium, copper, zinc, nickel and cobalt content. Comparison of heavy metal concentrations between table salt samples refined by recrystallization and washing methods is presented in Table 1. The values are reported based on dry weight. There were no significant differences between the two salt groups in lead, cadmium, copper, zinc and nickel content. Only the cobalt concentration was significantly higher in washed compared to the recrystallizad salts.

**Table 1:** Comparison of heavy metal concentrations between table salt samples refined by recrystallization and washing methods*

	**Recrystallized salts**		**Washed salts**		**P-value**	**Iranian food standard limits**	**Codex standard limits**
	**Mean ± SD**	**Range**	**Mean ± SD**	**Range**			
Lead (μg/g)	0. 309 ± 0. 263	0.021-0.921	0. 379 ± 0. 276	0.096-0.929	0.234	1.0	2.0
Cadmium (μg/g)	0. 020 ± 0.020	0.0026-0.078	0. 017 ± 0. 021	0.005-0.093	0.765	0.2	0.5
Copper (μg/g)	0. 114 ± 0. 064	0.026-0.234	0.198 ± 0.180	0.040-0.634	0.152	2.0	2.0
Zinc (μg/g)	0. 341 ± 0.228	0.049-0.829	0.376 ± 0.202	0.120-0.663	0.404	-	-
Nickel (μg/g)	0.152 ± 0.194	0.013-0.76	0.139 ± 0.233	0.009-0.920	0.284	-	-
Cobalt (μg/g)	0.008 ± 0.007	0.0002-0.026	0.037 ± 0.069	0.001-0.290	0.020	-	-

*Mann–Whitney U test.

Lead is a heavy metal that accumulates in the body and affects different systems and organs such as central and peripheral neural system, gastrointestinal tract, muscles, kidneys and hematopoietic system [27]. The maximum permitted level of lead in food grade salt is 2.0 μg/g according to the Codex legislation [28] and 1.0 μg/g according to the Iranian food standards [29]. In our recrystallized and washed salt samples, lead content was 0.30 μg/g and 0.37 μg/g respectively, which is below the permitted levels. In another report in Iran, lead concentration was 2.728 μg/g (range 0.01-5.8 μg/g) [9] and in salt samples from Tehran, lead content was 0.87 μg/g [16] and 0.438 μg/g [22]. In a study in Isfahan, lead content was determined to be 0.57 μg/g [23]. In the literature, it was reported in the range of 0.5-1.64 μg/g in refined and unrefined table salt samples from Turkey, Egypt and Greece [30] and 0.03 μg/g in Brazil [31].

Cadmium exposure induces bone damage, osteoporosis, and renal tubular dysfunction that leads to renal failure in long term [27,32,33]. It is also associated with several cancers [34-36]. In our recrystallized and washed samples, cadmium content was 0.02 μg/g and 0.017 μg/g, respectively. In other reports of Iran, cadmium content was 0.01-0.4 μg/g [9], 0.65 μg/g [16] and 0.024 [22] in salt samples from Tehran, and 0.15 μg/g [23] in Isfahan. In Brazil, cadmium content of salts was 0.01-0.03 μg/g [31], and in a study by Soylak et al., it was 0.014-0.030 μg/g in refined and unrefined table salt samples from Turkey, Egypt and Greece [30]. According to Codex legislation and Iranian food standards, the maximum permitted level of cadmium in food grade salt is 0.5 and 0.2 μg/g, respectively [28,29]. The cadmium content of our samples was less than these figures.

Copper is essential for good health, but its high intake leads to liver and kidney damage [9]. Based on Codex legislation [28] and Iranian food standards [29], the maximum permitted level of copper in food grade salt is 2.0 μg/g. In our samples, copper was 0.11 μg/g in recrystallized ones and 0.19 μg/g in washed ones, which is below the permitted levels, and as predicted, higher in washed salts than recrystallized type. Copper content was reported to be 1.21 μg/g [16] in Tehran and 0.87 μg/g [23] in Isfahan salt samples. In another report in Iran, copper concentration was in the range of 0.1-2.8 μg/g [9]. Copper content was 0.17-0.47 μg/g in Soylak’s study [30].

Similar to copper, zinc is an essential element at low concentrations, but in excessive levels, it has potential hazards to both animal and human health [37]. Zinc concentration was 0.34 μg/g in our recrystallized and 0.37 μg/g in washed salt samples. In Tehran and Isfahan salts, zinc content was reported to be 6.50 μg/g [16] and 6.34 μg/g [23].

Toxic effects of Nickel have been reported on the respiratory system, gastrointestinal tract, liver, and kidneys [21,38]. It also leads to neurological disorders [21,39]. In the only study in Iran, nickel content was reported to be 1.860 μg/g in refined table salt [21]. In our study, nickel concentration was 0.15 μg/g and 0.13 μg/g in recrystallized and washed salt samples, respectively. Soylac et al. reported the nickel content of refined table salt samples from Turkey, Egypt and Greece to be 0.16-1.57 μg/g [30].

Increased Cobalt level in the body negatively affects the fetus growth. It also interacts with physiologic actions of divalent metals such as calcium, magnesium, and biologic functions of vitamin dependent coenzymes [21,40]. In another study, cobalt concentration was determined to be 3.7 μg/g in refined table salts in Iran. In our recrystallized and washed samples, cobalt concentration was 0.008 μg/g and 0.037 μg/g, respectively. Cobalt content was 0.22-0.48 μg/g in Soylak’s study [30].

As we expected, lead, copper and zinc content was lower in recrystallized salts than the washed ones, but the difference was not statistically significant. Only cobalt content was significantly lower in recrystallized salts, compared to washed salts.

The calculated weekly intakes compared to their PTWIs are shown in Table 2. If people consume only recrystallized refined table salts, then we can say that their weekly intake of lead and cadmium is respectively 0.216 and 0.014 μg/kg body weight; If washed salt is used solely, then the weekly intake would be 0.2653 and 0.0119 μg/kg body weight for lead and cadmium, respectively, being far lower than the suggested PTWIs by the Joint FAO/WHO Expert committee on Food Additives (JECFA) [41]. By this consideration, people in Shiraz consume 0.8652-1.0612% of lead and 0.17-0.2% of cadmium PTWIs via salt consumption.

**Table 2:** Exposure to lead and cadmium through salt intake

**Heavy metals**	**Salt samples**	**Daily intake (μg/person)**	**Weekly intake (WI)**		**PTWI**	**(WI/PTWI) × 100**
			**(μg/person)**	**(μg/kg BW)**	**(μg/kg BW)**	**(% intake via salt)**
Lead	Recrystallized	1.854	12.978	0. 216	25*	0. 8652
	Washed	2.274	15.918	0. 2653		1.0612
Cadmium	Recrystallized	0.12	0.84	0.014	7**	0.2
	Washed	0.102	0.714	0.0119		0.17

*FAO/WHO, 1993 [41].

**Adapted from FAO/WHO, 2003 [42].

## Conclusion

All values for toxic metals were lower than the permitted maximum for human consumption as prescribed by Codex and ISIRI, and only 0.8652-1.0612% of lead and 0.17-0.2% of cadmium PTWIs are received via salt consumption weekly.

## Abbreviations

PTWI: Provisional tolerable weekly intakes; HDME: Hanging mercury dropping electrode; ASV: Anodic stripping voltammetry.

## Competing interests

The authors declare that they have no competing interests.

## Authors’ contributions

MHE helped in the design of the study and analytical phase. SMM participated in the design of the study, analytical phase, statistical analysis and drafted the manuscript. MA participated in the design of the study, analytical phase, statistical analysis and drafted the manuscript. MR helped in the analytical phase, and drafted the manuscript. All authors read and approved the final manuscript.
